# Practical considerations for transitioning early childhood interventions to scale: lessons from the Saving Brains portfolio

**DOI:** 10.1111/nyas.13684

**Published:** 2018-05-23

**Authors:** James M. Radner, Marvin J.S. Ferrer, Dominique McMahon, Caroline F.D. Black, Anuraj H. Shankar, Karlee L. Silver

**Affiliations:** ^1^ School of Public Policy and Governance University of Toronto Toronto Ontario Canada; ^2^ Grand Challenges Canada Toronto Ontario Canada; ^3^ College of Education Northern Arizona University Flagstaff Arizona; ^4^ Harvard T.H. Chan School of Public Health Boston Massachusetts

**Keywords:** early childhood development, scaling up social innovation, international development, program evaluation, implementation research

## Abstract

Small pilot studies of young children have frequently shown promise, but very few have been successfully scaled to the regional or national levels. How can we ensure that these promising approaches move from a suite of pilots to full‐scale implementation that can deliver sustainable impact for hundreds of millions of children? To elucidate concrete lessons learned and suggestions on accelerating the transition to impact at scale, we reviewed the Saving Brains portfolio to better understand three points: (1) the extent to which useful signals of impact could be extracted from data at the seed phase, (2) the ways in which innovators (project leaders) were approaching human resource challenges critical for scaling, and (3) the multisector diversity of the portfolio and the way innovators entered partnerships. The findings suggest key considerations for transitioning early childhood development interventions to scale and sustainability: strong entrepreneurial leadership, rigorous measurement and active use of data in support of adaptive learning, and champions acting at subnational levels. Together, these can enable flexible, iterative learning that can make the scaling process an opportunity to increase the level of benefit each child receives from an intervention.

## Introduction

The early years of a child's life are vital for brain development. The adversity children face during this critical window—including poverty, poor health and nutrition, and insufficient opportunity to play and learn—can disrupt normal brain development, causing setbacks lasting into adulthood. Globally, an estimated 43% of children are at risk of disrupted development due to extreme poverty and stunting, and at least one in three children fail to reach their full physical, cognitive, psychological, and socioemotional potential because of risk factors to early childhood development (ECD).^1^ More broadly, ECD is an essential element in building healthy, productive societies where children both survive and thrive, a reality that is recognized in the sustainable development goals and is central to the UN Secretary General's Global Strategy for Women's, Children's and Adolescents’ Health (2016–2030).^2^

Among the most effective ways to support ECD are interventions aimed at coaching parents or caregivers to interact with their young children in a responsive, emotionally engaging and cognitively stimulating way. These *psychosocial stimulation* 3, 4 interventions rely on improving interactions, behaviors, knowledge, beliefs, attitudes, and practices in parenting that lead to better psychosocial, motor, and cognitive development.^5^ Large‐scale delivery of such approaches is more complex than distributing a single product, since psychosocial interventions rely on human interaction, behavior change, and continuity of services across time. It is therefore not surprising that the field of ECD suffers from “pilotitis”: pilot studies of 10s to 1000s of young children have frequently shown promise, but few have scaled to the regional or national level (100,000+ children).^1^

Available experience in engaging large public systems to implement complex interventions suggests this challenge is substantial, especially in view of system capacity constraints, management issues, the need for effective local community engagement throughout the process, and resource limitations.^6^, ^7^, ^8^, ^9^, ^10^, ^11^ Public health systems in low‐resource settings have been able to reach scale with simple “point” interventions like vaccinations or vitamin A supplementation, but effective, high‐quality implementation of more complex interventions has proven elusive.^12^, ^13^, ^14^ For example, Bold and colleagues^15^ provide empirical evidence from a controlled scale‐up experiment of an educational intervention in Kenya, where public adoption of an evidence‐based program saw the effects fade into insignificance. These problems are not confined to low‐ and middle‐income countries; a U.S.‐focused meta‐analysis of 87 ECD studies found that “the largest effect sizes tended to have the fewest subjects.”^16^ Of those programs that have scaled, their impact is often limited by challenges in human resources management—for example, a lack of trained staff—that diminish quality at scale.^17^ A critical challenge, therefore, is how to ensure that the emerging suite of promising psychosocial interventions move from small‐scale pilots to full‐scale implementation in a way that can deliver sustainable impact for hundreds of millions of children.

Since 2011, Grand Challenges Canada and partners have brought attention to the importance of ECD through the Saving Brains program^18^: We source and support Integrated Innovation—projects that bring together social, business, and scientific/technological innovation^19^—that help children to thrive in their first 1000 days, from conception to 2 years of age. The vision of this program is simple: more children in low‐ and middle‐income countries reaching their full potential. To achieve this vision, Saving Brains supports and enables holistic solutions to develop and scale up products, services, and policies that protect and nurture early brain development in an equitable and sustainable manner.^2^ In particular, we help innovators make the process of transitioning to scale an opportunity to enhance their impact through flexible, adaptive learning, rather than to see impact degrade with scale, which is too often the result of simply duplicating validated interventions in expanded contexts.^20^, ^21^, ^22^, ^23^, ^24^, ^25^

A substantial literature on scaling up, both in social innovation generally^26^, ^27^, ^28^, ^29^, ^30^, ^31^, ^32^ and in the health sector^20^, ^25^, ^33^, ^34^ and ECD in particular,^35^, ^36^ offers a variety of insights and frameworks^37^ for application by program and policy leaders. Taken together, these frameworks suggest that scaling up is a multifaceted, multiphased leadership and management challenge, requiring attention to such diverse domains as: planning and strategy; organizational design; human resource management; policy, community and stakeholder engagement; monitoring and evaluation; adaptation of intervention content; building bridges between researchers and implementers; equity; and sustainability.^37^, ^38^ Any given intervention, in its own context, will need to develop plans, skills, and capacities across these diverse domains; there is no one‐size‐fits‐all approach.^32^, ^35^

This paper analyzes the Saving Brains portfolio to date, to better understand the lessons it has to offer on how to address these diverse challenges and accelerate the progress of psychosocial stimulation interventions toward sustainable impact at scale (See Box 1). As they develop psychosocial stimulation strategies, Saving Brains projects focus less on innovating around intervention *content*—where much is already known—and more on innovating for scalable, sustainable *implementation*. While all Saving Brains projects have learning agendas, our emphasis is on improving lives by scaling solutions that can be sustained at population levels, rather than on scaled‐up research per se.

**Box 1.**

**Key messages**

It is known that interventions focused on improving caregiver–child interaction can have substantial effects on young children's cognitive and socio‐emotional development. Many such interventions have been successfully piloted in low‐ and middle‐income countries, often with community‐level workers as frontline providers. There remains the challenge of how to scale the impact of such interventions at national levels in such countries. Early experience within the Saving Brains program demonstrates that it is possible to make progress toward overcoming this challenge by selecting and supporting a group of several dozen diverse projects with relatively short timescales and small‐to‐modest budgets. Saving Brains encouraged rigorous data collection on both process and outcome metrics, and engaged with project teams for shared learning. Resulting lessons highlighted the value of: using data for rapid‐cycle, adaptive scaling; mobilizing social entrepreneurs in service delivery; and partnering with on‐the‐ground stakeholders highly motivated to achieve impact at scale.

The Saving Brains portfolio is in no sense representative of interventions in ECD generally; it reflects the diverse, original qualities of its chosen innovations. Moreover, results are still preliminary, with different interventions in different stages of development and optimization. Therefore, we have not undertaken a standard meta‐analysis, nor have we tried to uncover new causal relationships. Rather, our perspective is that of a portfolio manager: We seek to derive and report lessons from our efforts to accelerate the progress of multiple, diverse innovations toward sustainable, large‐scale impact. Recognizing that there is no guarantee that our specific experience will apply in other settings without modification, our goal is to offer concrete suggestions or considerations that others can use, modify, and build on, as innovators and decision‐makers in ECD grapple with the transition to impact at scale.

We begin by providing further background on the Saving Brains program and its approach to the scaling challenges outlined above. We then describe the qualitative and quantitative methods used to review progress to date and derive lessons; present the results of that review; and discuss their implications. We close with a look to the future, including recommendations for action.

## Background

### The Saving Brains portfolio

To date, the Saving Brains program has invested ∼US$36.8 million in a portfolio of 84 innovation projects across 31 low‐ and middle‐income countries, and has enabled a platform of experts to support innovators^3^ in monitoring and evaluation, adaptive learning, and scaling. The portfolio includes pilot studies that are conducted with relatively modest resources (∼US$200,000) and in short periods of time (1.5–2 years), as well as a smaller number of larger projects (up to US$1.5 million over 1.5–3 years) making the transition to sustainable impact at scale. Priority areas for Saving Brains include: the first 1000 days of brain development; scalability in the public sector (enhanced by private sector and social entrepreneurship models, where beneficial); and equitable impact to children living in adversity and children traditionally left behind across large geographical areas.^4^ Taken together, the portfolio of innovative approaches to improve child development outcomes is achieving impact at the subnational level, and providing models for national and global change.

### The approach of the Saving Brains program to the challenge of impact at scale

At the heart of the Saving Brains approach to scale is a sense of urgency about *time*. A generation of children being born today are at risk, and we know^3^, ^5^ that in principle they can be helped. In practice, the scaling challenges reviewed above suggest that a great deal of adaptive learning will be required to translate theory into success for large populations of children. Thus, as each intervention team faces and resolves, phase by phase, its multiple management and delivery challenges,^20^, ^35^ collective progress will require rapid harvesting and sharing of resulting lessons. We do not have the luxury of time to first run carefully designed and controlled studies on 100–1000 children at a time to demonstrate incremental improvements in outcomes and, only after the results are in, to tweak the approach and try the next iteration half a generation later. Nor do we have ability to conjure enough resources to deliver to all at‐risk children interventions that depend on high‐cost strategies to achieve sufficient quality. The Saving Brains portfolio looked specifically for strategies that could be implemented in relatively short time periods in low‐cost settings, and for adaptive learning mechanisms that can cycle quickly and progress to impact at scale.

In managing and supporting this portfolio, our distinctive approach focuses on *both* impact *and* scalability at all stages of project development, rather than seeing the pilot stage as about impact only and dedicating later stages exclusively to replication. For example, one of the portfolio's transition‐to‐scale projects was based on a successful pilot where community health workers delivered a nutrition and psychosocial stimulation intervention that improved brain development in malnourished children in Bangladesh. Rather than simply replicate the pilot delivery model, which would have limited the number of malnourished children reachable by the existing cadre of health workers, the transition‐to‐scale project^5^ took advantage of the larger sample sizes to experiment with varying the delivery model. In place of one‐on‐one sessions with health workers and mothers, the project introduced group and paired sessions, and compared impact. The results pointed to a promising strategy, with the potential to serve fully the target population within available human resources.

**Figure 1:**
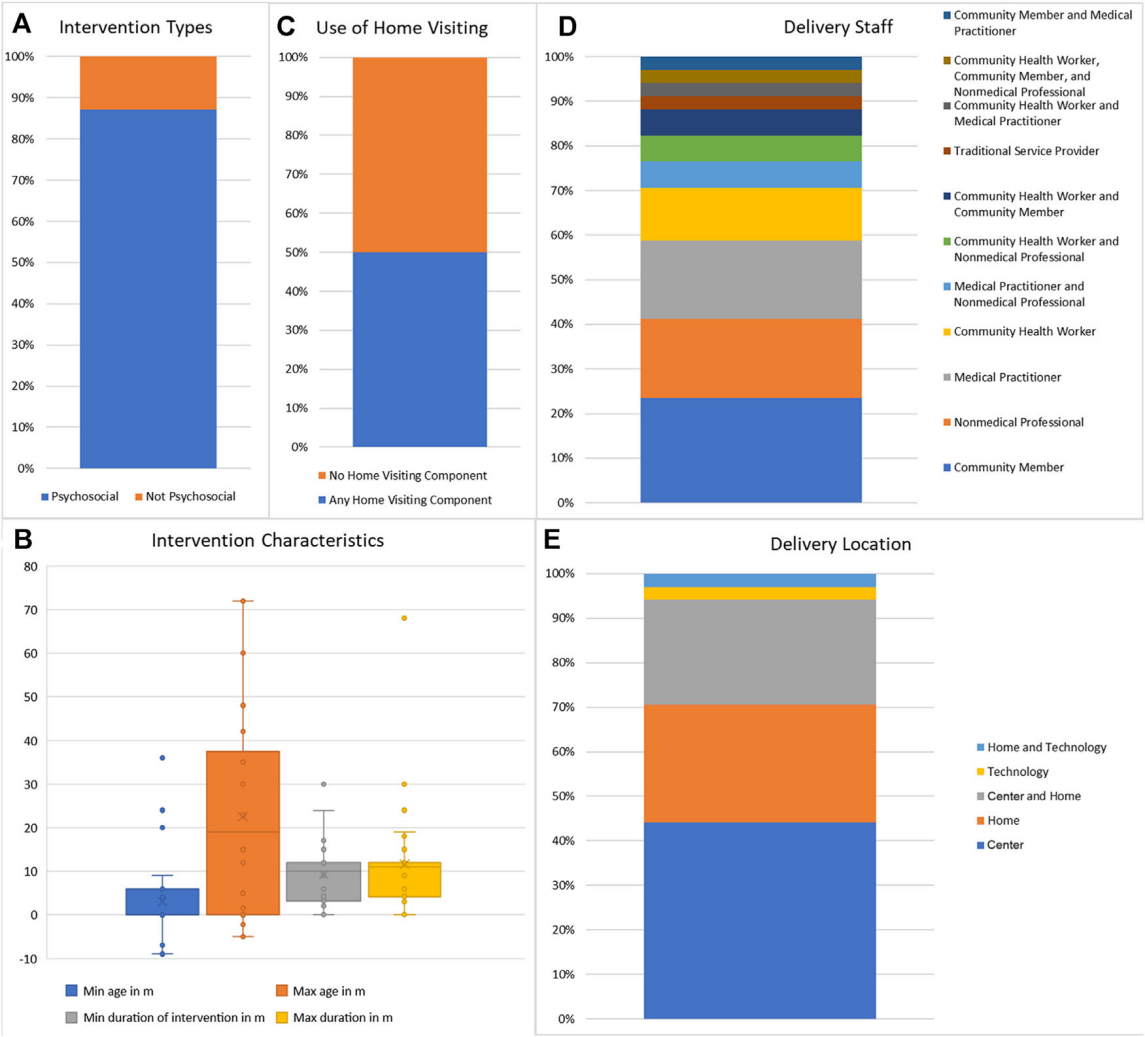
Descriptive details and characteristics of Saving Brains interventions. (A) The proportion with a psychosocial component, *n* = 39. (B) The minimum and maximum age of eligible children, and the minimum and maximum durations of the intervention for psychosocial interventions in the portfolio, *n* = 34. Interventions that began during pregnancy were counted as a negative age in months. (C) The proportion of psychosocial interventions with a home visiting component, *n* = 34. “Home‐visiting component” means the intervention included visits for intervention programming (and not merely for evaluation). (D) The proportion of different kinds of service providers delivering the psychosocial interventions, *n* = 34. (E) The proportional distribution of locations where psychosocial interventions were delivered, *n* = 34. “Center” includes a diverse set of locations such as childcare centers, clinics, hospitals, community centers, and other gathering places. “Technology” indicates the use, for example, of text messaging or video.

Our emphasis on rapid learning for impact at scale informed major design features of the Saving Brains portfolio, including:

- **Time frames**. Of the 39 Saving Brains projects completed by December 2016, the focus of this review, 34 were small “seed” projects with overall timing (including planning and reporting) of 1.5–2.0 years and five were larger “transition‐to‐scale” projects running 1.5–3.0 years. All five transition‐to‐scale projects and 29 seed projects included psychosocial stimulation. Within the psychosocial stimulation category, the median minimum service delivery period was 10 months, with 10.75 months the median maximum service period. Most of these interventions began at birth or before, or had no minimum entry age (Fig. 1).
- **Strategy**. Each project team developed a plan for impact at scale, with a 10‐year objective reflecting back to a concrete learning agenda for the current 1–3 year cycle. That learning agenda then informed the project's implementation, monitoring, and evaluation choices. It was during this impact‐at‐scale planning session, for example, that the Bangladesh project discussed above developed the idea of experimenting with service delivery through pairs and groups of mothers.
- **Measurement**. We encouraged project teams to collect rigorous data and track progress on each link in their theory of change, including implementation and service delivery milestones (e.g., baseline training and recruitment of families), intermediate outcomes (e.g., parent–child interactions), and ultimate child development benefits. Thus, Saving Brains projects all conducted both impact and implementation evaluation and monitoring. For this purpose, we provided specialized support on measurement selection and validation (through a team led by Penny Holding) and data management and analysis (led by Anuraj Shankar). Project teams reported on the full range of data semiannually to our portfolio management team, using a customized results management and accountability framework tied directly to each project's theory of change. This link enabled projects to investigate causal hypotheses, rather than report “black box” results. We also asked teams to look for differential effects—what works for whom—rather than only averages. Finally, we encouraged peer learning and supported webinars and workshops, so project teams formed a Saving Brains learning community.
- **Portfolio composition**. Each request for proposals called for Integrated Innovation,^19^ which enables scaling challenges to be informed not only by research, but also by practical experience from the public, social, and private sectors. We supported teams in developing strategic collaborations for scaling, with partners who could provide knowledge and capacity in addition to funding; we specifically sought engagement with both scientific researchers and social entrepreneurs. Social innovations make up the majority (72%) of the portfolio, while 20% are primarily business innovations, and 8% are primarily scientific or technological innovations. Most (62%) innovations were led by university and research organizations, with 28% by nongovernmental organizations (NGOs) and 10% by social enterprises.
- **Intervention delivery and content**. Psychosocial stimulation interventions were provided by cadres available at reasonable cost at the local level, generally community health workers, other community members, or nonmedical professionals; home visiting was a major delivery channel, but services were also provided in clinics, community centers, and childcare centers. Figure 1 breaks down the psychosocial interventions by service providers and delivery location; note the considerable heterogeneity on these dimensions. The Appendix (online only) provides summary sketches of each Saving Brains project involving psychosocial stimulation.

The Saving Brains program is still implementing these design principles through an ongoing, expanding portfolio. To synthesize lessons from the work through 2016, we reviewed and analyzed innovator experiences, as described below.

## Methods

### Themes and questions

Our purpose here is to synthesize and propose practical lessons from our ongoing work with the Saving Brains portfolio—lessons that can in turn be tested and refined, not only in our further Saving Brains efforts but also in other portfolios and initiatives. With this in mind, and noting that our role is that of a portfolio manager, not an independent evaluator, we conducted our analysis in the spirit of what Pritchett *et al*. call experiential learning^21^ (see also, Ref. 39), using mixed methods to refine and triangulate^40^, ^41^ our general observations. Since our portfolio is neither a representative sample of projects nor a large, homogenous set of studies, the quantitative aspect of our review uses descriptive rather than inferential statistics; we complement these with qualitative observations and interview results. The overall consistency of our results provides a measure of confidence that we are effectively describing the experience of *this* portfolio at *this* time; extending those results beyond the present context would require further investigation.

As a first step, we analyzed detailed notes (from two different note‐takers) and presentation slides from a workshop convened in Toronto in June 2016, where each transition‐to‐scale project team^6^ presented and discussed its strategy for sustainable impact at scale. We systematically reviewed this documentation and performed a cross‐case thematic analysis^40^ to synthesize major themes; once these were coded and extracted, the results were reviewed and checked (and adjustments made) by a facilitator who had attended all the sessions. We then used the themes that emerged as a basis for further analysis of the portfolio, as described below. At the highest level, these results coalesced around three priorities:

(1) The value of treating scaling as an adaptive process that fits both delivery mechanisms and intervention content to new contexts, with rigorous, ongoing measurement vital to assuring success;
(2) The centrality of human resource management to scaling psychosocial stimulation interventions; and
(3) The importance of in‐country stakeholder relationships and partnerships.

After the initial thematic review, we conducted a further qualitative and quantitative analysis of the Saving Brains projects, applying methods described below, to query, verify, and elaborate these themes and, bearing in mind our focus on combining impact and scale, to explore:

(1) The extent to which useful signals of impact, with the potential to support adaptive scaling, could be generated within the relatively short timetables and tight resource constraints implied by our seed grants;
(2) The ways in which our project teams were approaching human resource challenges critical to scaling; and
(3) The ways those teams were entering partnerships to support or deliver their interventions and how those experiences might hold lessons for successful transitions to scale.

### Respondent validation on scaling themes

The qualitative lessons reported here rely substantially on analysis of the notes and presentations from the June 2016 workshop described above. That workshop was held as the grant cycle for these projects was concluding, but before final reports were submitted. Subsequently, we reviewed the final reports against the initial analysis and interviewed each of the six project leaders (for approximately 1 h, generally with other team members participating), asking them to comment on, and propose changes to, the core themes, thereby completing a process of respondent validation.^41^ Participants received in advance a written synthesis of the “trial” themes and recommendations to be queried, and were encouraged to offer frank opinions, including disagreements, in the interview itself. One researcher conducted all six interviews; the interview guide began by requesting updates on recent experience and results and then queried, in turn, each “trial” item. All interviews were recorded, with participants’ permission. A second researcher reviewed the interviewer's detailed notes for accuracy against the complete recordings, with particular focus on participants’ views on “trial” items; the second researcher also reviewed the resulting synthesis in this paper in light of the audio recordings. Finally, a third researcher reviewed the validated interview notes against the material reported here.

### Analysis of “impact signals” from seed projects

All Saving Brains projects used a common, but customized, results‐based management and accountability framework to track both child development outcomes (physical, socioemotional, and cognitive development) and intermediate parental, parent–child interaction or home environment outcomes. Our purpose in analyzing those reported results was to assess the practicality of detecting signals on these dimensions on a short time scale and with modest investments—signals strong enough to guide iterative adaptation for scaling, even when the interventions themselves included the complexity inherent in behavior change efforts. Therefore, for this part of the analysis, we focused on the subset of completed projects that received seed funding to develop and test a psychosocial intervention. We reviewed and scored the measurement techniques and results for each of these metrics, based on reports from project teams at project close. These reports usually preceded full analysis for publication by team members, and our ratings are therefore far from summative or final assessments; in many cases, stronger or clearer results emerge once teams complete their work. Our lens, again, was that of a portfolio manager working on a pragmatic timetable.

With this in mind, we adopted a simple, four‐point scale to score early results on “*signal strength*” of reported outcomes, with “1” indicating little to no detectable signal and “4” referring to what we judged as a strong signal. We assigned to each project a signal‐strength score for its measured effects on child development outcomes, and a second signal‐strength score for home and parental outcomes. In each of these two domains, we regarded effect sizes of more than 0.2 standard deviations, significant at *P* < 0.05, as “strong,” generally meriting a rating of 4. We modulated this standard based on whether effects were sporadic across different measurements within the domain, or showed a logical pattern linked to the project's theory of change. Consistency within a domain, applied to significant effects of less than 0.2, was sufficient to earn a “strong” signal score, while a 0.2 effect size on an isolated measure would not yield a top rating unless the construct involved was the primary one being sought and measured for the relevant domain.

Projects were also scored 1–4 on measurement *quality*, with each project again receiving two scores, one for each domain. To roughly assess quality based on the reports from project teams, we considered whether the instrument used had been validated and found reliable in relevant contexts, whether it fit the construct involved, whether independent assessors were used where appropriate^7^, and whether controls (experimental or quasiexperimental) were in place.

Two raters scored all projects on all four metrics. There was substantial inter‐rater agreement (Cohen's weighted kappa 0.72), with virtually all discrepancies amounting to just one point on our scale. The raters then met and discussed each score, reconciling differences by consensus, based on information available from project reports.

Consistent with the origin and purpose of these ratings, our statistical use of them was descriptive. We report summary statistics along with a visual presentation; we also ran regressions, using Stata, as an aid to trend‐fitting, rather than to test any causal model. If measurements were usefully tracking the underlying dynamics from a project and portfolio management point of view, we would expect to see a positive relationship between signals of parent‐and‐home outcomes and child outcomes. Our analysis therefore queried whether that relationship existed, and whether it persisted when we controlled for measurement quality.

### Analysis of human resource requirements to deliver the interventions

We developed a simple form, used by all Saving Brains projects, to track human resource management costs and methods. For each worker cohort in their service delivery system, projects reported levels of education and professional qualifications needed, remuneration, baseline training hours and structure, supervision and refresher training, fidelity, and time required to deliver the intervention. Reports tracked both individual sessions and group sessions, including group size, to capture any related efficiencies. We reviewed the range of results across the Saving Brains portfolio and compared them with relevant data in published studies, which we coded in a consistent manner. We explored the variation in the data by performing k‐means cluster analyses on domains of human resource practice relevant to the challenge of high‐quality, cost‐effective scaling, covering educational qualifications, place‐of‐service delivery, baseline and refresher training, supervision, and group and individual service dosages.^8^ We shared our descriptive results with Saving Brains project teams at a joint session in October 2016, using the comparisons to spark a thematic discussion of key factors in human resource management, and integrated the results of that discussion into the qualitative, thematic review described above.

Finally, we compared training hours and service dosages for the Saving Brains psychosocial stimulation seed projects based on their “signal strength” ratings for child development outcomes, with reference to the coded published studies. Here, as throughout our analysis, our approach was descriptive rather than inferential, given the nature of the small, heterogeneous sample of Saving Brains projects and our goal of deriving pragmatic lessons.

To identify comparison studies, we reviewed the literature through keyword and reference searches, and screened the resulting articles to prioritize experimental or quasiexperimental interventions that included psychosocial stimulation delivered by nonprofessional, para‐professional, or practice‐based workers, and that measured cognitive, social, and emotional outcomes for children under five. We coded 58 such studies and used them for the comparisons described here. These coded studies, which covered a substantially wider range of parameters than those focused on here, could also form the core of a larger meta‐analytic database that could in turn support future statistical analysis beyond the scope of the present paper. Coders received substantial initial and refresher training, with regular joint sessions to prevent “drift.” We conducted random reliability testing and assured that all studies were double‐ (sometimes triple‐) coded. Inter‐rater agreement percentages ranged 0.80–0.92; a senior coder resolved all discrepancies.

### Partnerships and their role in scaling strategies

Under the overall rubric of Integrated Innovation,^19^ Saving Brains project teams were recruited from diverse sectors and encouraged to enter cross‐sector partnerships in the design, testing, and scaling of their interventions. We reviewed the presentations and discussions of partnerships from the June 2016 Toronto meeting and the final reports of the transition‐to‐scale projects, and looked for qualitative lessons from examples of partnerships that were achieving traction in moving from the pilot level to scale. We then tested our conclusions via the respondent validation interviews described above.

## Results

### Thematic review

The respondent validation interviews strongly reinforced the core themes that form the backbone of this paper and generally supported our explicit “trial” recommendations, which we reformulated to take account of respondent comments. (See our concluding section.) Respondents all saw scaling as an adaptive process, because of new contexts—for example, different cultural or community settings—and because of differences in implementation structure required at larger scales. Respondents underscored the importance of relationships in human resource management, and highlighted the complex relationships they needed to develop and maintain with diverse local and national partners. Relationships with governmental partners were crucial but introduced challenges due, for example, to turnover in key positions and slow‐paced decision‐making. Again, there was no “one‐size‐fits‐all” strategy, but rather a need to try different approaches and make fit‐to‐purpose modifications as problems emerged.

Particular emphasis in these follow‐up interviews (even more than in the original data) centered on the challenge of maintaining quality while scaling. Respondents saw monitoring as vital, and use of quality and fidelity tools as potentially helpful, but not in a rote way: rather, to secure quality, teams aimed to identify and maintain major “active ingredients” essential for success (e.g., that service providers engaged responsively with parents when delivering curriculum), even as they adapted intervention details (e.g., curriculum specifics and delivery protocols) to new contexts. Monitoring outcome data as well as process data helped assure that intended benefits were sustained or enhanced as the intervention evolved.

Cost constrained the effort to maintain quality. Project leaders had to make trade‐offs and look for cost‐effective solutions, for example, to human resource and data collection challenges. The strategies and vignettes reported below, for example, creative uses of technology, can thus be seen as adaptive responses by Saving Brains innovators to the ongoing challenge of delivering high‐quality psychosocial interventions at increasing scale and feasible cost. Teams often then found themselves acting as custodians of quality for larger systems, providing training, monitoring and evaluation, and capacity‐building for local systems. Collecting appropriate data was central to this effort; the review that follows begins with our experience with outcomes metrics.

### Quality and magnitude (“signal strength”) of outcomes metrics

Our results reporting framework recognizes that direct measurement of child outcomes is needed to understand the impact of potential approaches. Predictive measures or leading indicators for child development outcomes are also needed to speed up the iteration of interventions, at both piloting and scaling stages, for better impact and scalability. Other efforts are underway (i.e., Bill & Melinda Gates Foundation's Healthy Birth, Growth and Development initiative^42^) to develop new predictive measures (e.g., imaging of brain development). Meanwhile, we have used metrics based on the caregiving environment (e.g., measures of parent–child interaction) as “leading indicators” of possible impact for psychosocial interventions. Our review explored the feasibility of this approach in the low‐resource, fast‐cycle context of our seed grants, to see whether strong and consistent signals could be detected in both the “leading” (caregiving environment) and child developmental categories.

**Figure 2:**
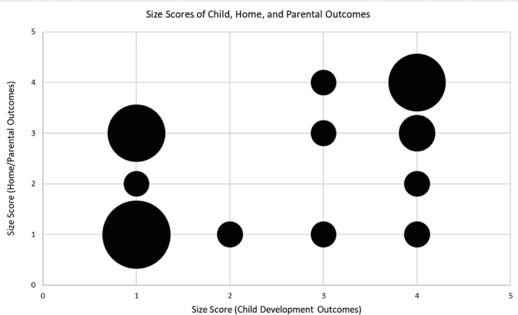
The relationship between signal strength size scores for child development outcomes (horizontal axis) and home/parental outcomes (vertical axis) is shown. Larger bubbles mean more projects with those scores.

Analysis of signal‐strength scores showed no statistically significant difference between mean scores for child development outcomes compared to home and parental outcomes, on both the measurement quality and signal‐strength (effect size) scales. Measurement quality scores were generally good for both categories (median 4 for developmental outcomes, and 3 for home and parental outcomes), but the difference in median signal‐strength scores (1.5 versus 3; see Table 1) suggests that projects were more able to demonstrate substantial effects on home and parental outcomes than on child outcomes.^9^ Still, many projects (10 of 29) were able to show sizable and statistically significant effects on child development within the 2‐year period of seed funding. As Figure 2 illustrates, projects with strong signal‐strength (3–4) scores for child outcomes generally also had strong signal‐strength scores for home and parental outcomes, but a cluster of projects (upper left in Fig. 2) had poor scores on the former but good scores on the latter.

**Table 1:** Descriptive statistics for quality and signal‐strength scores for child development outcomes, and home and parental outcomes

	Quality score of child development outcomes	Quality score of home/parental outcomes	Signal‐strength score of child development outcomes	Signal‐strength score of home/parental outcomes
Median score	4	3	1.5	3
Interquartile range	1	2	3	2

Regression analysis contributed to our evaluation of reported signals by assessing whether the expected, logical relationships between the signals across the two domains (child versus caregiver‐home outcomes) held, and whether the relationship was diluted when controlled for quality. The results were encouraging (Table 2): a 1‐point increase in the signal‐strength score of home and parental outcomes was associated with a 0.60‐point increase in the signal‐strength score for child development outcomes (*P* = 0.001). When we controlled for the quality of measurement for both child development and caregiver‐home outcomes, the correlation between signal‐strength scores persisted, but shrank slightly from 0.60 to 0.53 points (*P* = 0.006). The change in the signal‐strength score of child development outcomes associated with a 1‐point increase in the quality score of child development outcome measurement was 0.43 (*P* = 0.033). The quality score of home and parental outcomes did not have a significant effect on the signal‐strength score of child development outcomes. Overall, the combination of child and parent‐home outcomes used by our seed portfolio emerges as a promising way to detect signals of impact in the short term, and thus to support the kind of iterative development needed, we suggest, for cost‐effective impact at scale.

**Table 2:** Regressions: the dependent variable is the signal‐strength score of child development outcomes

	Signal‐strength score of home/parental outcomes	Quality score of child development outcomes	Quality score of home/parental outcomes
Signal‐strength score of child development outcomes	0.60 (0.001)	–	–
Signal‐strength score of child development outcomes (with controls)	0.53 (0.006)	0.43 (0.033)	–0.16 (0.40)

Ordinary least squares coefficients are reported with *P* values in parentheses.

### Human resources analysis

Human resources are often scarce and stretched in high‐poverty areas, yet the ability to strengthen the parent–child interaction depends on effectively training, managing, and supporting such resources. Our human resources analysis explored how far human resources currently stretch to achieve meaningful and significant outcomes. We found considerable heterogeneity across projects and noted that the Saving Brains portfolio was extending the range of practices typical of the published studies (e.g., by providing, in general, higher levels of in‐service supervision). We found salient clusters in human resource practices, suggesting that project teams were optimizing training, supervision, and delivery approaches, depending on both intervention context (cultural factors and available resources) and intervention content. Thus, while there was no universal answer to such questions as how to balance baseline and follow‐up training hours, the portfolio as a whole was engaged in adaptive learning to discover different strategies to fit multiple niches.

We shared clustered data with innovators so that they could see where their practices were similar to or diverged from their peers, and then discussed the reasons for their distinctive choices. Innovators responded by highlighting efforts to improve relationships between families and frontline workers, and between frontline workers and the rest of the system. These efforts were largely based on problem solving and challenging social norms that limit child development outcomes. For example, frontline worker training in a project in Ethiopia^10^ included discussions of challenges to the traditional gender norms of family care; this prepared workers to support fathers as active participants in early childcare. A project in Jamaica^11^ aimed to reduce stress of parents with children with sickle cell disease by integrating a parenting‐and‐play program with components designed to enhance problem solving and stress management.

In cases where technology was used to extend human resources, we observed that the technology was most powerful when it allowed the frontline worker to focus on relational elements of the exchange with parents, while still delivering high‐quality content. In Vietnam, group parenting sessions, or women's clubs, begin with a short video presentation followed by worker‐led discussions and role‐play activities, providing opportunities for participants to practice new skills.^12^ Similarly, technology was effectively used to present standardized information for further discussion in projects in Ethiopia and Rwanda,^13^ and further experimentation is beginning in several new projects just joining the Saving Brains portfolio. Results from a completed mHealth project in Brazil^14^ suggest that even low‐touch mobile applications that have not demonstrated direct child development benefits can reinforce positive parenting knowledge and practices, and improve care‐seeking behavior, potentially acting as a powerful component layered on other higher touch interventions. Finally, our transition‐to‐scale projects reported using technology, generally to support relationships in the service chain (e.g., through WhatsApp chat groups) and sometimes to support training (through videos, either to record sessions and provide immediate feedback, or to provide presentations) or diagnostics.

**Figure 3:**
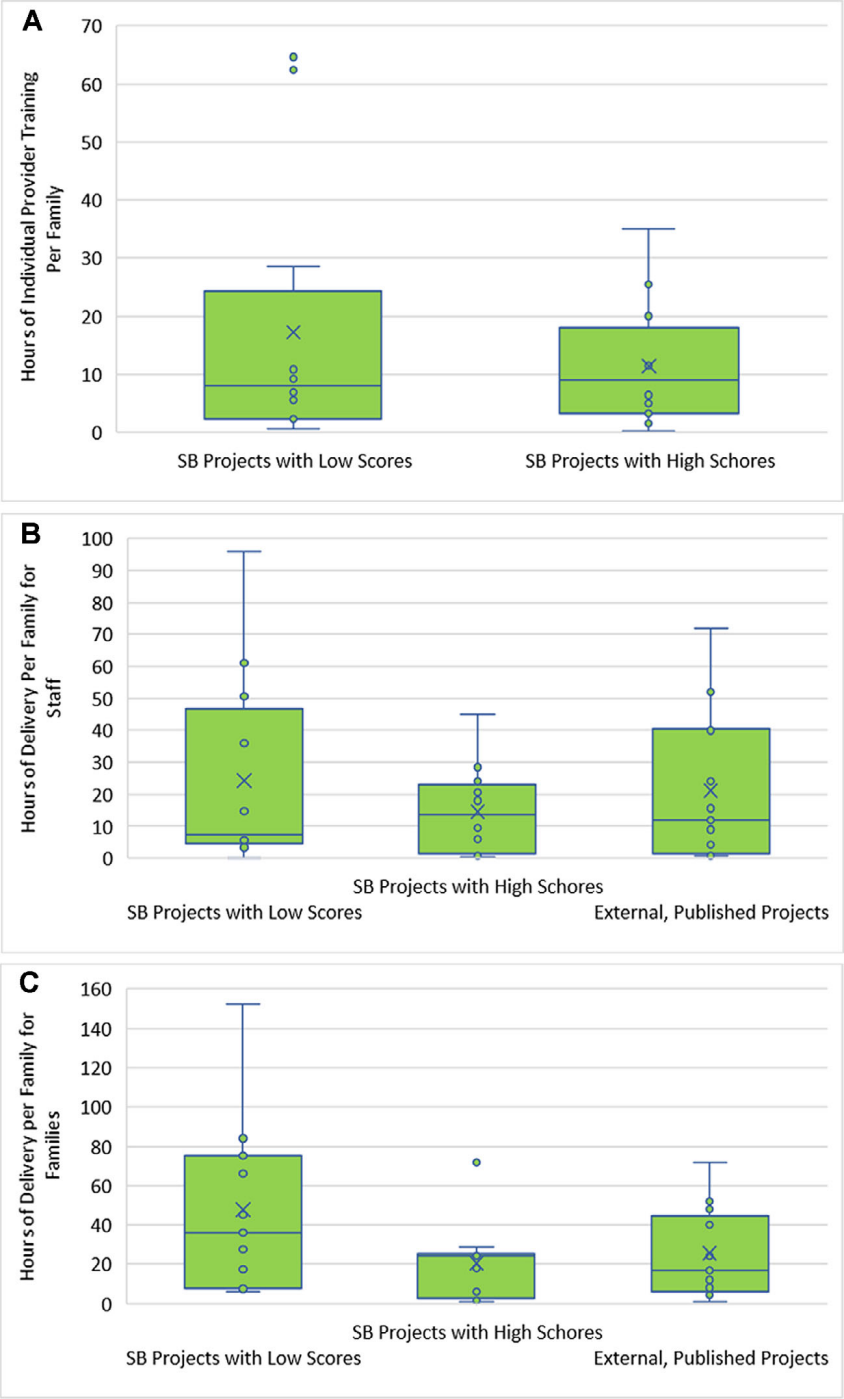
Relationships between human resources burdens in proof‐of‐concept psychosocial “seed” grantees and child outcome size (signal strength) scores, where data were available. Saving Brains projects with child development signal‐strength scores of 1 or 2 (SB Projects with Low Scores) were compared with those with scores of 3 or 4 (SB Projects with High Scores), as well as external, published studies we coded (External, Published Projects). (A) Hours of training for an individual staff member per family served are shown, *n* = 24. Training includes initial baseline training, refresher training, and supervisory time. There was an insufficient *n* for published literature (*n* = 3). (B) Hours spent by staff delivering interventions, *n* = 38. Two outliers were removed. (C) Hours spent by families receiving interventions, either in groups or individually, *n* = 38.

The heterogeneity of the Saving Brains projects—reflected in both the cluster analysis and the content of the interventions—and the preliminary, pragmatic nature of the signal‐strength outcomes we reviewed prevented us from specifying and testing a full causal model on effects of human resource decisions. Instead, Figure 3 provides a descriptive picture of the key delivery variables across the projects based on their child outcome effect‐size ratings, with comparisons to the way those variables ranged in the published studies we coded. To calibrate the training investment, we calculated total training (baseline and refresher) plus supervision hours service providers received, and divided by the number of families they served; for dosage, we separately considered both provider time (which could be in groups) and client time (itself a cost) per family served. There is no clear relationship between outcome score and the per‐family parameters; instead, the picture is again heterogeneous, especially among the Saving Brains projects. (The lower impact Saving Brains projects exhibited more spread in the level of human resource investment, but this does not imply that such investment is unproductive.) Different context‐dependent and intervention‐specific factors likely drove the decisions of project leaders on human resource investments and intervention doses, given resource constraints. For example, group delivery is a way to reduce both training and service delivery time per family served; in the Bangladesh example described above^15^, group sizes were determined by space limitations in small rural clinics, rather than by any “universal” estimation of ideal size. Questions of optimal group size, and group‐individual trade‐offs, remain an important area for inquiry and context‐based learning.

### Project teams and partnerships

Saving Brains projects work with a range of scaling partners, including governments, foundations, NGOs, and private sector enterprises. Some of the scaling partners attracted by Saving Brains innovations act nationally. For example, a university‐based project^16^ aiming to improve government child services^17^ in rural Colombia has maintained a long‐standing research‐based collaboration with the national government, with the result that data emerging from the project have influenced major government investment in ECD.

We also have observed strong local leadership, coming primarily at the subnational and municipal levels. For example, the municipality of Carabayllo, Peru and a local NGO are collaborating to scale a community‐based innovation for children at risk of developmental delays.^18^ With strong mayoral support, the municipality is providing funding from its Participatory Budget, where allocation is decided by local citizens and civil society. We have observed that in‐country demand for projects approaching transition‐to‐scale—as signaled by the mobilization of local finances and the emergence of an influential champion—can be especially salient at municipal or regional levels. Since other municipalities in Peru have shown interest in Carabayllo's example, this model also represents a promising approach to sustainable scaling. Meanwhile, we saw that motivated local partners can help solve implementation and context adaptation challenges inherent in scaling up psychosocial interventions.

As they transitioned to scale, the Peru project team shifted its role to emphasize training and capacity‐building with service providers and collaboration with community agencies. Another transition‐to‐scale project, Mobile Crèches,^19^ is also scaling through capacity‐building with local partners, in their case working with NGOs to provide quality crèche services in India. In Bangladesh,^20^ the scaling step now underway involves “training‐the‐trainers” with a substantial group of rural, government‐run community health clinics. At an international level, Reach Up^21^ and Kangaroo Mother Care^22^ are both successfully acting as “centers of excellence,” providing training and expertise to support implementation in multiple countries (Brazil, Guatemala, Jamaica, and Zimbabwe^43^; and Cameroon and Mali). See Appendix (online only) for project descriptions.

Finally, we noted that social entrepreneurs in the portfolio tended to innovate continuously in their delivery models, even in early stages, before questions of how to optimize intervention content were sorted out. For example, one social enterprise worked to develop a daycare model at an affordable price‐point in low‐income settings,^23^ with a network of both formal and informal providers. Through iterative changes in the program and through engagement with parents and teachers, they achieved and demonstrated quality improvements at both the formal and informal centers, thereby creating a platform to combine impact and sustainable scalability.

## Discussion

### Limitations

Our analysis was based on the internal structure of the Saving Brains portfolio, and thus depended on the distinctive profiles of a small number of heterogeneous projects. This inherently limits generalizability of our findings, as does our focus on descriptive statistics and qualitative review. The lessons derived from this kind of review are best seen as part of a larger experiential learning process in the broader early childhood field, so that portfolio managers, innovators, and stakeholders—including Saving Brains itself, as we expand our portfolio—can try out, build on, and modify our recommendations, based on further results. Thus, our findings represent a contribution to the kind of broader learning enterprise recommend by Shonkoff *et al*.^44^

In that spirit, we have worked to harvest specific, focused lessons from the Saving Brains portfolio, rather than comprehensive solutions. For example, we focus on using human resource management to assure high‐quality delivery, but there are many other relevant quality challenges encountered even within our own portfolio, for example, logistical problems in remote settings. Moreover, human resource management itself is a large subject, to which we offer only a limited contribution here. The ongoing learning platform hosted by the Early Childhood Workforce Initiative^45^ is exactly the type of broader learning enterprise to which we hope our lessons can contribute; cf. their recent report on training.^46^ Another major human resource topic that our projects confronted is staff turnover. While we hope our approach to enhancing management–staff and staff–client relationships can help reduce turnover, this is a hypothesis that requires testing.

We have noted that scaling questions admit no “one‐size‐fits‐all” answer. This admonition applies to efforts to use, in any given context, the insights and approaches described here. For example, our focus has been on interventions that work with caregivers to enhance their relationships with young children; it is natural that such interventions are not readily reducible to a small number of concrete, easily, and universally replicable steps. Other types of interventions—for example, public campaigns and medical checklists—have a different character and may be better suited to scaling by strict replication. Massoud *et al*.^47^ provide examples, though even here they note that “constant adjustment and adaption will be required” (p. 21).

### Reflections

The challenge facing the field of ECD is to rapidly and effectively scale approaches that best enable children to thrive. Our analysis of the Saving Brains portfolio sheds some light on actions that may accelerate this process and enable scale to actually facilitate, rather than hamper, improved outcomes. In particular, by asking innovators to take an integrative view of challenges that are too often considered separately—challenges in optimizing impact and in reaching scale sustainably—we found substantial grounds for hope. Approaches that harness rigorous measurement of intervention results to strong social entrepreneurial leadership can support adaptive learning on how to achieve higher effect sizes at feasible cost in multiple settings. Such approaches can address in a unified way the need to optimize intervention content, manage stretched human resources, and partner effectively with large funding and delivery systems.

Our emphasis on rigorous measurement recognizes that measurement is costly. We observed project teams working to embed measurement capacity in local communities, with local participation in developing not only intervention content but also measurement strategy. We have seen that this is a promising approach for both efficiency and sustainability.^48^, ^49^ We advocate careful measurement of implementation variables, intermediate outcomes (“leading indicators”), and final child development outcomes; budgetary and practical constraints will cause projects to select measurement priorities based on the distinctive learning and monitoring needs they face at any given point in the scaling process and on key questions relating to their theory of change. Thus, measurement strategy, for both choosing priorities and building capacity, is itself an aspect of adaptive scaling. In this light, we encourage portfolio managers and project teams, including researchers, to recruit social entrepreneurs into the effort to cost‐effectively embed and scale measurement.

If ECD innovators see their role as identifying and designing solutions for markets reaching millions of families, they will need to set up, from the beginning, a system that allows ongoing empirical learning while scaling. As shown by the composition of the portfolio, few social entrepreneurs have rallied to the Saving Brains challenge, yet their leadership capacities are needed to tackle some of the biggest persistent challenges around scale and sustainability. At the core of entrepreneurship lies the capacity to scale. In contrast to academics, who define or apply leading‐edge knowledge, entrepreneurs design for a market and keep adapting until that market is satisfied. It is important to note that adaptability is not the same as lowering rigor. In fact, data‐driven adaptation has the potential to increase rigor for solving the scaling challenge.

Emphasizing data and evaluation—even at early pilot stages—can also help to quickly identify potentially promising approaches that merit further exploration by innovators and further investment by funders. Continued rigorous experimentation at the scaling phase can help solve key delivery challenges, as our Bangladesh example shows. We validated our hypothesis that meaningful and significant improvements in home and parental outcomes that are more proximal to the intervention are more likely to be detected than corresponding improvements in child development outcomes. The more proximal, leading indicators are not to be confused with having validated predictive power—still the holy grail for ECD work. Instead, they represent a consistent and reliable way to rapidly understand the potential effect of an intervention on the caretaker–child dyad and support iterative learning as the project (justifiably) attracts more resources.

For example, a seed project in Vietnam^24^ was able to show that their women's clubs innovation improved home environment scores and maternal mental health, plus a range of other secondary outcomes (breastfeeding, child health, father's engagement in play, and knowledge of dangerous symptoms during pregnancy), even though child outcomes approached but did not reach statistical significance. So compelling were these results that Australia's National Health and Medical Research Council is funding a longer, larger randomized controlled trial to determine impact. The team is also using these secondary outcomes to identify which modules seemed most effective and which could use further iteration. In this way, sourcing widely for potential approaches to ECD can be used to relatively rapidly (1.5–2 years) and inexpensively (up to US$200,000) select innovations that are then worth investing larger amounts of money to transition to scale.

At the same time, we were buoyed by the number of pilot projects that were able to measure meaningful and significant child outcomes. Because such outcomes often cannot be detected until late in the project cycle, measuring them is less helpful in guiding necessary iterations during the project period, but important to ensuring ultimate success. Note that diversity within populations means that there is great variability in the individualized impact of interventions, due to differences in individual adversities or biology, including genetic and epigenetic variations within populations. This was well demonstrated by Morgan *et al.*,^50^ where the effect size of a maternal–infant attachment intervention increased by more than 2.5‐fold for individuals with a certain genotype of serotonin transporter polymorphism, and reduced by 10‐fold for those with another genotype.

While we have not measured genetic indicators within Saving Brains, we do encourage project teams to track individual differences, and to focus on especially vulnerable children. We are also observing population‐wide large effect sizes without subgroup analyses when an intervention adopts such a focus, for example, with malnourished children in Bangladesh.^25^ Understanding what works for whom, even in small pilots, can better tease apart the potentially substantial effect on sensitive individuals from a population effect that often yields small‐to‐moderate average effect sizes.

In sum, rigorous data at early stages in intervention development are valuable for far more than a binary answer to a once‐and‐for‐all question: Does it work? Instead, data can provide signals that contribute to an iterative quest for improved impact based on the question: What is working, for whom, in what contexts, and why? From this point of view, scaling represents an opportunity to improve impact rather than see it degrade: Larger numbers enable both discovery and application of insights about differential effects, with a goal of improving impact for all vulnerable children.

The dominant scaling paradigms in ECD yield high quality only at high cost. The current trade‐off between quality and cost needs to be an area of active exploration for the field. This exploration should primarily be aimed at optimizing limited human resources, to focus on what leads to the most impact. The ability to gather meaningful outcome data within short time periods, as just reviewed, is critical to any adaptive learning effort to solve human resource challenges. Attention to such results can then go hand in hand with a direct empirical focus on relevant human resources data. Our review of the Saving Brains portfolio identified different strategies for developing human resources systems that seemed promising individually; when combined, these may hold even more promise for transitioning to scale interventions to support parent–child interactions in stretched, low‐resource delivery settings. For example, Saving Brains innovators frequently engaged service systems to meaningfully interact with many families, and to continuously adapt based on both leading indicators of the quality of the interaction (e.g., behavior change that leads to improved nurturing care) and the number of families served.

Such strategies require good human relationships within service systems, and may benefit from innovations under development in the portfolio that deploy technological solutions to facilitate connections between service providers and client families. These might include digital applications or text‐based communications that can be disseminated on mobile phones—to find clients, to provide simple, replicable content about the “active ingredients” of nurturing care, and to track encounters to ensure continuity. Among our project teams in developing countries, we observed high usage levels of mobile phones, and related applications, including various forms of messaging. We suggest that continuing and expanding low‐cost availability of such technologies represents a significant opportunity for ECD when seen as way to support and enhance, rather than to replace, high‐quality human relationships.

A necessary part of the scaling equation is strong demand from in‐country champions for ECD. From observations about the domestic champions who are effectively “pulling” interventions from the Saving Brains portfolio to scale, we propose identifying significant local partners (such as municipalities, districts, or NGOs with geographical reach) as starting points for scale. Cities—or sizeable rural districts where a single agency or NGO has coverage capacity—can provide a strong foundation for national scaling. Local units with sufficiently large population and with their own leadership can offer an opportunity to learn (and continuously adapt) at scale, and provide a compellingly relevant demonstration for other localities. This is in contrast to the dominant, country‐level scaling approach (e.g., Chile and India),^5^ which has found limited global success. Country‐level support may be necessary but not sufficient, since a national “supply push” relies on policy and politics, with limited ability to optimize for quality.

### Path forward

Building from this analysis, we offer three practical recommendations for innovators and decision‐makers who are fueling progress for ECD globally:

(1) Measure child development outcomes at every phase of testing and scaling, but design pilot studies to include a focus on at least one strong intermediate outcome (e.g., parent–child interaction) that is found in the theory of change and can be used to inform rapid‐cycle, adaptive learning as the intervention scales. Researchers have a leading role to play in this action.
(2) Design human resources systems with capacity to adapt to context, assuring quality delivery and community responsiveness, as the intervention scales. Apply technology to facilitate (rather than replace) high‐quality relationships between frontline workers and families, and between frontline workers and their supervisors. Social entrepreneurs have a leading role to play in this action.
(3) Conduct initial scaling efforts in partnership with motivated local stakeholders with significant on‐the‐ground reach, such as municipalities, large rural districts, or locally active NGOs or corporations, engaging with sufficient client populations to assess and adapt the intervention for impact at scale. This can complement national‐level support and enable local demand to energize scaling. Local champions have a leading role to play in facilitating this action.

Finally, we suggest to managers of intervention portfolios—including, in the case of Saving Brains, Grand Challenges Canada and its partners—that there is great opportunity in applying, further testing, and building upon lessons of the kind identified here. Doing so will require continued attention to rigorous, rapid data collection, as well as shared experiential learning with on‐the‐ground participants. We look forward to a learning journey with all who wish to take up this challenge.

## Competing interests

The authors declare no competing interests.

## Supporting information

Appendix Tables S1 and S2.

## Footnotes

1 “Pilots” can themselves be at different scales, such as those serving 10s versus 1000s of children and, in some contexts, a “scaled up” project naturally refers to what we refer to as a larger pilot. Since our purpose is to explore impact at the regional and national population levels, in this paper we reserve the term “scale” for those levels; we refer to projects reaching the level of 1000s of children as pilots undergoing “transition to scale.”

2 This is aligned with the scientific literature on the integrated concept of “nurturing care.”^5^

3 All Saving Brains projects are selected for their innovative value to the field; thus, we refer to Saving Brains project leaders and team members as “innovators.” These can include researchers, program developers, business or social entrepreneurs, or members of civil society organizations.

4 Because Saving Brains projects currently operate at subnational scales and frequently target regions or populations where adversity (e.g., malnutrition) is high, the portfolio does not generally classify its projects based on national‐level, population‐wide statistics, such as the Human Development Index (HDI). For example, Saving Brains has worked on scaling up an intervention targeted for malnourished children in Bangladesh, though Bangladesh as a whole is a medium human development country as measured by HDI.^52^

5 Led by Jena Hamadani, International Centre for Diarrhoeal Disease Research, Bangladesh. www.savingbrainsinnovation.net/projects/0463-03/.

6 The six participating teams included all five transition‐to‐scale projects completing by December 2016 (listed in the first table in the Appendix, online only), plus a sixth project, *Socios en Salud Sucursal Peru*, which completed its seed phase in that period (and therefore appears in the second table in the Appendix (online only), among the seed projects, and in our quantitative analysis) and was beginning the transition to scale at the time of the workshop.

7 For example, self‐reports are generally appropriate to measure parental stress levels or depression, and parental reports are often used to assess children's socio‐emotional development, but independent observation is helpful for parent–child interaction metrics.

8 The coding and analysis were carried out for Saving Brains by a team that included Anuraj Shankar, James Radner, Elizabeth Prado, Caroline Black, Purnawati Rachman, Indriya Laras, and Nicholas Graham.

9 The relative ease of detecting home and parental outcomes is natural and familiar in the literature across a wide range of contexts. (For a recent review in a high‐income setting, see Ref. 51.) Our purpose here was to examine whether useful and logical signals could be derived in the context of small, short‐cycle seed projects in low‐resource settings.

10 Led by Philip Tanner and Tirussew Teferra, Christian Children's Fund of Canada, Ethiopia. www.savingbrainsinnovation.net/projects/0589-03/.

11 Led by Jennifer Knight‐Madden and Susan Chang‐Lopez, The University of the West Indies, Jamaica. www.savingbrainsinnovation.net/projects/0584-03/.

12 Led by Tuan Tran and Thach Tran, Research and Training Centre for Community Development, Vietnam. www.savingbrainsinnovation.net/projects/0344-03/.

13 Intera za Mbere. Led by Richard Ashford and Caroline Dusabe, Save the Children, Rwanda. www.savingbrainsinnovation.net/projects/0724-03/.

14 SMS Bebê. Led by Juliano Froehner and Michael Kapps, TNH Digital Health, Ltda., Brazil. www.savingbrainsinnovation.net/projects/0579-03/.

15 Led by Jena Hamadani, *op. cit*.

16 Led by Raquel Bernal, Universidad de los Andes. www.savingbrainsinnovation.net/projects/0462-03/.

17 The Family, Women and Infancy program.

18 Led by Leonid Lecca and Llubitza Maribel Muñoz Valle, *Socios en Salud*, Peru. www.savingbrainsinnovation.net/projects/0769-05/.

19 Led by Mridula Bajaj, Mobile Crèches for Working Mothers’ Children, India. www.mobilecreches.org/.

20 The work led by Jena Hamadani, *op. cit*.

21 Led by Susan Walker and Christine Powell, University of the West Indies, Jamaica. www.reachupandlearn.com/.

22 Led by Nathalie Charpak, Fundación Canguro/Kangaroo Foundation, Colombia. http://fundacioncanguro.co/.

23 Led by Sabrina Premji and Racheal Nduku, Kidogo Early Years, Kenya. www.savingbrainsinnovation.net/projects/0578-03/.

24 Led by Tuan Tran and Thach Tran, *op. cit*.

25 The project led by Jena Hamadani, *op. cit*.
