# Supplementary material for: Practical considerations for transitioning early childhood interventions to scale: lessons from the Saving Brains portfolio
Source: Ann N Y Acad Sci. 2018 May 23;1419(1):230–48. doi: 10.1111/nyas.13684 (PMC9764263; doi:10.1111/nyas.13684)
Supplement: Supplementary file 1 — Appendix Tables S1 and S2. [file NYAS-1419-230-s001.docx]

**Appendix**

**Project-by-project summary tables for selected portions of the saving brains portfolio**

The tables below present summary sketches of (1) the five Saving Brains transition-to-scale projects that finished by the end of 2016 and (2) the 34 psychosocial stimulation seed grants finishing by the same date. *Seed grants* support the development and validation of innovative ideas to protect and nurture early brain development, offering up to $250,000 CAD over a maximum of 18 months. By the end of the grant, projects were expected to demonstrate proof of concept that the innovative idea is likely to have sustainable impact at scale, i.e., provide on-the-ground evidence of the impact it may have on an individual child and how it may be feasibly implemented in the target region. *Transition-to-scale* grants support the refinement, testing and implementation of innovative solutions that have already achieved proof of concept to bring them toward scale, offering up to $1 million CAD in matched funding over a maximum of three years. Projects were expected to demonstrate real-world impact on early brain development, with an approach that increases the intervention reach and has strong potential for sustainable impact at scale.

The “number of children reached” column in the tables refers to members of the treatment group (i.e., this number excludes controls). Fuller descriptions of all projects can be found at [www.savingbrainsinnovation.net/](file:///C:\Users\James%20Radner\AppData\Local\Microsoft\Windows\Temporary%20Internet%20Files\Content.Outlook\FHOW5A7D\www.savingbrainsinnovation.net\).

| *Project Title* | *Institution Name* | *PI Name* | *Implement-ation Country* | *Project Summary by Innovator* | *# of Children Reached* | *Setting* | *Service Provider Cadre* |
| --- | --- | --- | --- | --- | --- | --- | --- |
| *Saving Brains; Changing Mindsets* | *Mobile Crèches* | Mridula Bajaj | *India* | *Improving child development by training NGOs to deliver well-established, free, holistic childcare program for young children of migrant construction workers. The primary focus of the project is to build an entire management framework to run these crèches, in partnership with current and new builder partners, and service-providing NGOs, and transferring the business operations.* | *4,845* | *Urban* | *Non-Medical Professionals; Community Health Workers; Community Members* |
| *Reach Up: Expanding access to a proven early stimulation program through a web-based package and technical support* | *The University of the West Indies* | *Susan Walker/ Christine Powell* | *Brazil, Zimbabwe, Guatemala and Jamaica* | *A home-visiting program aimed at supporting parents of 0- to 4-year-olds to improve their child’s development through play. Materials include a curriculum of activities, a toy manual, a structured training course for home visitors (training manual with demonstration videos), a guide for supervisors, and an adaptation manual and planning guide.* | *206* | *Rural; Urban* | *Community Members* |
| *Transition to scale of an integrated program of nutritional care and psychosocial stimulation to improve malnourished children's development in Bangladesh* | *International Centre for Diarrheal Disease Research, Bangladesh* | *Jena Hamadani/Fahmida Tofail* | *Bangladesh* | *Integrating a cost-effective evidenced-based program of psychosocial stimulation into community clinics and examining the effect on malnourished children’s development through two cluster randomized controlled trials.* | *1,597* | *Rural* | *Medical-Practitioners* |
| *Randomized Open Controlled Trial on Kangaroo Mother Care versus Traditional Care for Low-Birth-Weight Infants: Patient-Centered Outcomes at the Age of 18 years* | *Fundación Canguro / Kangaroo Foundation* | *Nathalie Charpak* | *Mali; Cameroon* | *Improving global diffusion of KMC by building a culturally sensitive train-the-trainer model supported by an e-learning and data tracking platform, implemented initially in Cameroon and Mali, where infant mortality rates are among the highest in the world.* | *2,385* | *Urban; Peri-Urban* | *Community Members; Medical Professionals* |
| *An Integrated Intervention Targeted at Deprived Pre-School Children in Rural Colombia* | *Universidad de los Andes* | *Raquel Bernal* | *Colombia* | *Incorporating a structured early stimulation curriculum and a nutritional intervention into public national parenting support services for vulnerable families living in rural areas of Colombia.* | *2,134* | *Rural* | *Non-Medical Professionals (educator)* |

| *Project Title* | *Institution Name* | *PI Name(s)* | *Country* | *Project Summary* | *# of Children Reached* | *Setting* | *Service Provider Cadre* |
| --- | --- | --- | --- | --- | --- | --- | --- |
| Learning clubs for women’s health and infant development | Research and Training Center for Community Development | Tuan Tran/  Thach Tran | Vietnam | Women's Learning Clubs seeking to improve cognitive, social-emotional and motor development of young children, and to reduce wasting rate in children under 24 months. | 175 | Rural | Community Members |
| Fathers Involvement: Saving Brains in Vietnam | Hanoi School of Public Health | Tran Bich/Lynn Rempel | Vietnam | Improving child development by promoting engaged and effective father-infant interactions and father supported breastfeeding. | 361 | Peri-Urban; Rural | Community Members |
| An Integrated Toolkit to Save Newborns’ Brains | Hospital for Sick Children | Shaun Morris/  Robert Armstrong | Kenya | A portable toolkit containing evidence-based tools that reduce the incidence of neonatal insults (including newborn mortality and poor brain development). Users learn how to effectively stimulate their newborns through education on newborn stimulation messaging involving play, responsive caregiving and communication strategies. | 1,572 | Rural | Community Health Workers |
| Improving Early Childhood Development in Zambia | Zambia Center for Applied Health Research and Development | Davidson Hamer/  Godfrey Biemba | Zambia | A cluster-randomized trial of 30 clusters of villages to test the feasibility and impact of a package of community-based early childhood services in a rural area of Southern Province, Zambia. Two main services: 1) biweekly visits from a local health worker, screening children for infections and malnutrition; and 2) biweekly mothers’ group meeting where caregivers were taught a diverse parenting curriculum covering cognitive stimulation and play practices, child nutrition and cooking practices, and self-care for good mental health. | 259 | Rural | Community Members |
| Effect of community-based depression management and psychosocial stimulation intervention on maternal mental health and child development: a randomized controlled trial | International Centre for Diarrhoeal Disease Research, Bangladesh (icddr,b) | Fahmida Tofail | Bangla-desh | Combining an innovative model of Cognitive Behavioural Therapy for mothers with mild-moderate depression (including parental coaching on psychosocial stimulation), with an evidence-based play-stimulation ECD intervention for children. | 142 | Rural | Non-Medical Professionals |
| Community-based Family Coaching for Children with Developmental Risks in Lima, Peru | Socios En Salud Sucursal Peru | Leonid Lecca/  Llubitza Maribel Muñoz Valle | Peru | Delivered by community health workers (CHWs) to infants 6–24 months experiencing or at high risk of neurodevelopmental delay, the intervention provides high-quality training and supervision of CHWs on the delivery of community-based interventions for these infants. Children and their primary caregivers were enrolled and randomly assigned to one of three arms: 1) monthly nutritional support; 2) nutritional support plus three months of CBI delivered in the home; or 3) nutritional support plus three months of CBI delivered in group settings. | 41 | Rural | Community Health Workers |
| CommCare ECD: Mobile Technology and Community Health Workers for Early Childhood Development | Ugunja Community Resource Center | Aggrey Omondi/  Charles Ogada | Kenya | Two cost-effective mobile apps (one for community health workers and one for caregivers) to enable each to support and assess early child development in home-based settings. The apps offer users practical advice, tools, educational aids and forms for assessing and fostering early childhood development, including: cognitive development, nutritional support, management of common illnesses, and counseling on cognitive stimulation for parents and caregivers. | 313 | Rural | Community Health Workers |
| Proving concept on a sustainable and scalable model of Early Childhood Care and Education for urban slums in Kenya | Kidogo Early Years | Sabrina Premji/  Racheal Nduku | Kenya | Kidogo Early Years is a social enterprise that seeks to transform the trajectory of young children by providing high-quality, affordable, early childhood care and education in urban slums. | 435 | Urban | Non-Medical Professional (educator) |
| Novel SMS-Based Technology for Early Brain Development Support and Monitoring in Brazil | TNH Digital Health Ltda. | Michael Kapps/  Juliano Froehner | Brazil | An interactive SMS-based engagement program received on parents’ cellphones that can be used to educate and remind parents of best practices for parenting and interactions that promote early childhood development, and to monitor the development of their children from prenatal through to the infant’s third month. | 393 | Urban | Medical Practitioners |
| Community-based infant massage: from saving bones to saving brains | CEL Ventures Private Limited | Aarti Kumar | India | Integrating a package of early child development interventions into the existing socio-cultural practice of infant massage provided by community massage specialists and families that is universally prevalent in low-resource settings. The intervention involves targeted home visits, as well as community mobilization, as part of the behaviour change management approach. | 777 | Urban; Rural | Community Health Workers |
| Family-Inclusive Early Brain Stimulation (FInE BrainS) | University of Ibadan College of Medicine | Akindele Adebiyi/ Oladimeji Olayinka | Nigeria | Using existing post-natal/child welfare clinics to deliver an intervention that uses culturally acceptable videos and active skill building, to deliver health messages and practical skills to women on early child development. Mothers will, in turn, train their male partners/other caregivers within the context of the home setting. | 480 | Urban | Medical Practitioners |
| Fostering the development of premature babies through stronger child-family well-being: innovating early child development assessment and intervention in high-risk infants and their families in Brazil | Fundação de Desenvolvimento da Pesquisa | Claudia Alves/ Lívia Magalhães | Brazil | Newborn Behaviour Observation introduced into the routine of the Casa do Bebê/Sofia Feldman Hospital (Brazil), as a means to help parents understand their babies´ needs and how to deal with them. Each mother/family will participate in workshops to build a portfolio on their babies. On each follow-up visit (five per family/baby during the first year), families will participate in small workshops using their own records on the portfolio to talk about their feelings, the baby´s achievements and how to promote their development. | 53 | Urban | Medical Practitioners; Non-Medical Professionals (social workers) |
| Home visiting programs to improve early childhood development and maternal mental health - evidence from the Western Region Project | Fundação Faculdade de Medicina (FFM) | Alexandra Brentani/  Sandra Grisi | Brazil | A bi-monthly home visiting program by a childcare worker to support mothers in providing child health, development and stimulation. Exploring two delivery platforms: 1) A newly trained cadre of Child Development Agents for children receiving traditional centre-based care; and 2) integration of home visiting programs into the Family Health Strategy performed by Community Health Agents | 319 | Urban | Non-Medical Professionals |
| Integrating a Parenting Intervention with Routine Care to Improve Early Developmental Outcomes in Children with Sickle Cell Disease and Decrease Maternal Stress | University of the West Indies | Jennifer Knight-Madden/ Susan Chang-Lopez | Jamaica | Integrating a stimulation and education package into the routine care of children with sickle cell disease, composed of modules and videos, augmented by lessons in problem solving, for parents to better cope with medical and psychosocial challenges of caring for a child with this chronic condition. | 32 | Urban; Rural | Medical Practitioners |
| A sustainable public-private partnership for delivering integrated child development care in Pakistan | Association for Social Development (ASD) | Muhammad Khan/ Shazia Maqbool | Pakistan | Engaging private clinics to promote optimal early development of young children in poor urban environments, through an integrated package of mother and child care. The care package will have three main components: information on nutrition, information on child development, and screening and management of maternal depression. | 1,037 | Urban | Medical Practitioners |
| A community-based implementation of a low-cost, evidence-based toolkit for improving brain development in newborns who suffered neonatal insults | The University of Manitoba | James Blanchard/  Tahira Reza | Pakistan | Combining evidence-based interventions with a parenting program to create an intervention package delivered by link workers alongside birth attendants. The year-long parenting program is implemented by the link workers and parents, and covers developmental skill areas, such as cognitive and fine motor, social and self-help and gross motor skills, aimed at augmenting brain development among newborns, including those neonates who might have suffered a neonatal insult at birth. | 447 | Rural | Traditional Service Providers |
| A Community-based Conscious Discipline Program to Reduce Corporal Punishment in the Caribbean | Windward Islands Research and Education Foundation Grenada, Inc. | Randall Waechter/  Barbara Landon | Grena-da | Training in “Conscious Discipline” for Grenada’s pre-existing Roving Caregivers (Rovers), who traditionally deliver infant stimulation in the absence of a traditional caregiver. The Rovers help parents learn positive, “brain smart” discipline and parenting techniques to foster neurodevelopment in young children by focusing on safety, attachment and self-regulation, in addition to stimulation. | 843 | Peri-Urban; Rural | Community members |
| Maternal and Newborn Health and Early Childhood Development in Rural Low-Literacy Settings of Ethiopia | Christian Children’s Fund of Canada | Philip Tanner/  Tirussew Teferra | Ethiopia | "Learning through Play” program, using audio-visual materials, including locally adapted pictorial calendars depicting the successive stages of child development, with brief descriptions of simple play activities that show parents what they could do to promote healthy child development. Delivered through Ethiopia’s national health extension program. | 3,000 | Rural | Non-Medical Professionals |
| Readiness to Learn in the Early Years: Learning Lessons for Scale | The Aga Khan University | Muneera Rasheed/  Aisha Yousafzai | Pakistan | Evaluating the impacts of a program called LEAPS [Youth Leaders for Early Childhood Assuring Children are Prepared for School], in which youth manage the use of community-based preschools and conduct community engagement strategies. | 215 | Rural | Community Members |
| Scaling early childhood development at Anganwadi Centers in India | Datta Meghe Institute of Medical Sciences (Deemed University) | Abhay Gaidhane/  Prakash Behere | India | Training Anganwadi workers (community workers providing child health and early learning services) to deliver an enhanced curriculum. Through this positive parenting program, the intervention hosts both group parent meetings and home visits for vulnerable families. | 875 | Rural | Community Health Workers; Non-Medical Professionals |
| Crowdfunding and social networks as a novel mechanism to sustainably promote physical growth and positive psychosocial and neuro-developmental outcomes in severely stunted Guatemalan children | Wuqu'Kawoq Maya Alliance | Peter Rohloff/ Maria del Pilar Grazioso | Guate-mala | An innovative crowd-sourcing financing mechanism to support a home-based nutritional intervention targeted at “catching-up” rural children’s nutritional and development outcomes, which complements other existing preventative nutrition programs. | 298 | Rural | Non-Medical Professionals; Community Health Workers |
| Matuzo Bora Ya Watoto Wachanga: Good Care for Babies | Heartland Alliance International | Constantin Kahorha | Congo Dem. Rep. | Improving infant development outcomes through providing mental health services to mothers experiencing major depression, due to exposure to human rights violations and sexual violence. The program relies on government-funded healthcare facilities, and trains existing community health workers to deliver a culturally adapted, evidence-based intervention for depression. | 157 | Rural | Medical Professionals; Non-Medical Professionals |
| The effects on early brain development of a nurse home visitation program for pregnant youth and their families living in a poor urban area in São Paulo, Brazil | University of Sao Paulo | Guilherme Polanczyk/  Euripedes Constantino Miguel Filho | Brazil | The effects on early brain development of a nurse home visitation program for pregnant youth and their families living in a poor urban area in São Paulo, Brazil | 34 | Urban | Medical Professionals |
| Strengthening Health Systems for High-Risk Newborn Care: An Integrated Hospital-Community GIS System for Sustainable Delivery of Quality Care during Early Child Brain Development in Rural Uganda | Makerere University | Gertrude Namazzi/ Darius Kajjo | Uganda | Improving healthcare for high-risk babies in Uganda, utilizing community health workers to identify high-risk babies and refer them to an appropriate health facility for care, through the use of “FOOT length cards” and “Danger sign screening cards”. Existing community health workers are trained to identify and register pregnant women and mothers, and conduct three home visits in the first week of birth: on the day of birth, three days post-birth, and seven days post -birth. | 203 | Rural | Community Health Workers; Medical Professionals |
| First Steps (Intera za Mbere): Promoting healthy early childhood development by extending holistic parenting education nation-wide through radio-facilitated peer learning groups, and by increasing access to emergent literacy materials | Save the Children Canada | Richard Ashford/ Caroline Dusabe | Rwanda | Combining radio programming with community-based peer learning groups, the First Steps program includes four components: 1) educational radio programming; 2) a package of materials, including a facilitator’s guide, activity cards, and a children’s book; 3) a trained area facilitator to support local volunteers to lead parent sessions and conduct home visits; and 4) interventions to increase the availability of books and learning materials. | 1,620 | Rural | Community Members |
| A community-based model of delivery of Kangaroo Mother Care for improving child survival and brain development in low-birth-weight newborns | Society for Applied Studies | Sunita Taneja/  Nita Bhandari | India | Community Kangaroo Mother Care (CKMC): a low-cost intervention delivered at home to reach low-birth-weight babies in a sustainable way. Aims to prove that CKMC will result in better newborn and infant survival; growth and development in low-birth-weight babies, through better breastfeeding rates; better growth measures; and less episodes of childhood illnesses. Mothers who perform KMC expected to show higher bonding and attachment, and less maternal depression. | 276 | Peri-Urban; Rural | Community Health Workers; Community Members |
| Steps Brain Booster Program | Steps Baby Lounge | Glaucia Maciel/ Daniel D. Santos | Brazil | A replicable and low-cost coaching, training and lesson plan methodology, with state-of-the-art equipment and toys, that can enable local educators and caretakers in crèches to improve the quality of stimulation offered to children during school hours. | 305 | Urban | Non-Medical Professionals (Day-care Staff) |
| Integrating early child development in home-based environmental interventions in rural Peru | Universidad Peruana Cayetano Heredia | Stella Hartinger | Peru | Combining early child stimulation intervention (ECS) and an integrated, home-based environmental intervention package (IHIP) to homes in rural Peru. The ECS intervention consists of simple activities identified as crucial to promote children’s development and mother’s mental health, e.g., through increased caregiver-child interaction, and increased play with age-appropriate toys and communication. A household-environmental health intervention package (i.e., improved cooking stoves, kitchen sinks and hygiene education IHIP) has been implemented to address the state of the direct environment in which young children develop. | 236 | Rural | Community Members; Community Health Workers |
| Chamas for Change: Building Adult Capabilities to Safeguard Children’s Developmental Potential through Mother-Child Clubs in Kenya | Governing Council of the University of Toronto | Astrid Christoffersen-Deb/Julia Songok | Kenya | Integrating a new parenting curriculum into existing “Chamas”, peer support groups that have been successful at improving maternal and child health outcomes in previous studies. | 613 | Rural | Community Health Workers |
